# Athletes’ and Coaches’ Perceptions of Nutritional Advice: Eating More Food for Health and Performance

**DOI:** 10.3390/nu13061925

**Published:** 2021-06-03

**Authors:** Danielle M. Logue, Laura Mahony, Clare A. Corish, David Tobin, Ronan Doherty, Grainne O’Higgins, Sharon M. Madigan

**Affiliations:** 1 Sport Ireland Institute, Sports Campus Ireland, Abbotstown, D15 PNON Dublin, Ireland; lmahony@instituteofsport.ie (L.M.); dtobin@instituteofsport.ie (D.T.); ronan.doherty@lyit.ie (R.D.); gohiggins@instituteofsport.ie (G.O.); smadigan@instituteofsport.ie (S.M.M.); 2 Physiotherapy and Sports Science, School of Public Health, University College Dublin, D04 V1W8 Dublin, Ireland; clare.corish@ucd.ie; 3 Sports Lab North West, Letterkenny Institute of Technology, F92 FC93 Letterkenny, Ireland

**Keywords:** low energy availability, health, challenges, benefits

## Abstract

Background: Low energy availability results in physiological adaptations which contribute to unfavourable health outcomes. Little information exists on perceptions of nutritional advice to eat more food to maintain health and enhance performance. The aim of this study was to explore athletes’ and coaches’ perceptions towards advice to athletes to eat larger than their current quantities of food and to explore how nutritionists could deliver this advice. Methods: Semi-structured interviews (~20 min in length) were conducted using online communication technology, audio-recorded, and transcribed verbatim. The interview explored perceptions of the nutritional advice provided, its role in health and performance, and the challenges to eating larger amounts of food. Data were analysed using NVIVO 1.2 using an inductive thematic approach. Results: Nine elite athletes (female = 6; males = 3) and nine high-performance coaches (female = 3; male = 6) completed the semi-structured interviews. Athletes reported improved training consistency, fewer injuries and illnesses, and improved resilience when consuming adequate energy and nutrients to meet their needs. Lack of time and meal preparation difficulties were the main challenges faced to fuelling. Conclusions: Although education about under-fuelling is important, motivating, enabling, and supporting athletes to change behaviour is pivotal to increasing athlete self-awareness and to make long-term nutritional changes.

## 1. Introduction

Having sufficient energy available (energy availability, EA) for basic physiological functions can positively influence health and performance [1,2,3]. In 2014, relative energy deficiency in sport (RED-S) was described by the International Olympic Committee (IOC). RED-S refers to low EA (LEA) and can occur in both male and female athletes [3]. LEA is defined as having insufficient energy for normal physiological function after the energy cost of exercise has been removed [1]. LEA/RED-S may have unfavourable health effects on body systems as well as negatively impacting on athletic performance [4,5,6]. Hence, it is vital to optimise both short- and long-term nutrition strategies to reduce risk of injury and illness, and to improve training adaptation and performance [3].

Adequate energy intake is essential for maintaining normal reproductive function and bone health [6,7]. LEA can arise from disordered eating behaviours, clinical eating disorders, the timing of training conflicting with eating opportunities, or lack of education [2]. Independently or in combination, these can make taking sufficient energy to meet training and performance demands difficult to achieve on a daily basis [8,9,10]. Furthermore, external pressures (body image, and family/sport culture) from others (family, coaches, and fellow athletes) can be a contributory factor for LEA/RED-S. On the other hand, coaches and other sports professionals can positively influence athlete wellbeing by reinforcing the message around fuelling and by facilitating regular screening for risk of LEA using an appropriate tool, for example, the Low Energy Availability in Females Questionnaire (LEAF-Q) in females [11]. Although LEA risk has been investigated in elite male cyclists [12], no agreed screening tool for males exists and the development of such a tool is warranted.

To the best of our knowledge, there are no data available on the perceptions of athletes and their coaches towards advice to consume more energy to support health and training/performance demands. The purpose of this study, therefore, was to explore athletes’ and coaches’ perceptions towards advice to eat more food to fuel health and performance as well as the challenges that athletes face in following this advice. A secondary aim of the study was to explore how nutritionists could deliver this advice to achieve greater impact.

## 2. Materials and Methods

### 2.1. Participants

In order to determine the viability of the interview questions for use with athletes and coaches, interviews were conducted with two coaches who were not included in the final project. Two questions were removed following this pilot as feedback from the two coaches was that the time taken to complete the interview needed to be reduced. The questions removed did not substantially impact on the information to be collected. The final semi-structured interview included the following categories: (1) background information and participant characteristics; (2) benefits and challenges of adequate fuelling; (3) application and delivery of message on fuelling; and (4) gaps in education. Using email, fliers and video calls, elite athletes and high-performance coaches were invited to complete the semi-structured interview online during June and July 2020. Sporting organisations under the auspices of the Irish national sports governing organisation, Sport Ireland, were contacted and asked to inform athletes and coaches of the study. Participant inclusion criteria were as follows: (1) being advised to consume more calories by performance nutritionist (athlete); and (2) working with athletes who were advised to consume more calories (coach). The interviewer (DL) was available by email to clarify any questions from participants about the interviews. The study was approved by the Human Research Ethics Committee of University College Dublin (LS-20-36-Corish). All subjects provided informed consent online before completing the interview.

### 2.2. Procedure

All interviews were conducted by a research nutritionist (DL) using Zoom (Zoom Video Communications, California, USA) as a result of COVID-19 social-distancing requirements and were audio-recorded. Interviews lasted approximately 20 min. A semi-structured topic guide with open-ended questions designed to create discussion on topics relevant to the study was used. Prior to the interview, information was collected on: (1) participant demographics; (2) type of sport/coaching; (3) number of hours spent exercising/coaching per week; (4) perceptions about increasing calorie consumption (i.e., understanding, feelings, and advantages/disadvantages of eating more); and (5) behavioural change (i.e., how the advice has been perceived over time, what supports athletes/coaches need to implement recommended dietary changes, and advice for sports practitioners working with athletes at risk of LEA).

### 2.3. Data Analysis

All interviews were transcribed using Rev (Rev transcription company, 2010, Austin, TX, USA), then analysed using NVivo 1.2 (QSR International, London, England). An inductive thematic analysis procedure was applied to the data to identify common themes. Transcripts were read repeatedly by a second author, L.M., who was blinded to the participants, and initial trends within the data generated. Both D.L. and L.M. independently and systematically coded the transcripts, and then discussed the codes to verify their application to the data. Overarching themes and sub-themes were identified, and quotations were extracted to illustrate typical views.

## 3. Results

### 3.1. Participant Characteristics

Nine elite athletes (female = 6; male = 3) and nine high-performance coaches (female = 3; male = 6) completed the semi-structured interviews. All participants were engaged in sport at an elite level and had access to nutrition services through Sport Ireland Institute (Table 1 and Table 2). The mean number of hours athletes spent training each week was 27 (±7). The mean number of hours coaching per week was 16 (±10). The mean athlete age was 26 (±3) years.

### 3.2. Benefits and Challenges of Adequate Fuelling

The sub-themes and representative quotes associated with the benefits and challenges of adequate fuelling are presented in Table 3.

#### 3.2.1. Benefits

Six coaches and two athletes identified an improvement in the consistency of training resulting in better performance as a major benefit of fuelling adequately. Participant 12 described how adequate fuel allows for consistency in training:

“The big one is energy. With swimming, it’s a huge load within a week. It’s a lot of training, even at a club level. And I’m coaching teenagers, they do anywhere of up to 14 h, excluding gym work. So actually, they could do up to 16, 17 h training a week. So for me, it’s just that they’re able to give as much as they can at each session” (Participant 12—Coach).

Four athletes and three coaches perceived that they had better immune function and were able to avoid recurrent injuries/illnesses as another benefit of fuelling for performance:

“Now I felt like, once I got my head around, I was way better, had way more energy. My mood was better. I was in better form. My immune system was much better. I wasn’t getting sick as much. Yeah. It all had a huge impact obviously then on my performance as well. It was really good” (Participant 15—Athlete).

Participant 9 emphasised the importance of maintaining optimal health for consistent training and performance:

“Health is the primary one. If someone is consistently falling ill, that’s a big issue, and it will often manifest through upper respiratory type infections. Athletes present what may be cold or flu-like symptoms, or just general getting stale, not progressing their training. They might just reach a bit of a brick wall before they can move on. With some others, there can be underlying health issues as well when it comes to bone density, bone health” (Participant 9—Coach).

Furthermore, a number of athletes reported having more energy available which improved training quality and resilience at training camps:

“Just my recovery, which is really nice. From then, I could feel the massively positive impact that had; my resilience on training camps” (Participant 3—Athlete).

#### 3.2.2. Challenges

Five coaches and five athletes acknowledged that the main challenges to adequate fuelling were time management around training and food preparation in this study:

“Time is a huge thing. I work 20 h a week in the school, but it’s going to training, going to work, coming home. And that’s for four or five days in a week. It’s driving all over the place. So timing is a huge thing. I have to be super prepared. If I’m not, I fall off the wagon. For me, the day before, I’d have to have everything prepared. The dinner is the only thing I’d prepare when I come in. You never get to relax, because all you’re doing is cooking and eating and training and moving. And that is quite difficult” (Participant 15—Athlete).

The financial burden and the volume of food required were identified as challenges to eating adequately to support training demands by coaches and athletes:

“But I think us not having the capacity or budget in terms of providing meals, I think that is a barrier. Whether people like to admit it or not. Because you can say all the right things and provide athletes with all the right information but if they have to stop in a garage and get a sandwich or a wrap or whatever, you’re still going to have a percentage of the time where they’re not going to make the right choice” (Participant 2—Coach).

“It’s just very difficult to eat that quantity of food. Even when I did the pre-prepared meals, but if I got one of those big ones that was like 900 calories, I’d have to eat half of it and then come back to half of it a little bit afterwards. It’s just difficult to eat a large quantity. And my stomach didn’t feel that great eating a huge, huge amount” (Participant 14—Athlete).

The participants also identified the risk of gaining weight as a challenge to eating adequately:

“I think I was just paranoid about gaining weight. It was my main thing that I struggled with. And even I weighed myself and I got heavier, but I thought I looked a lot leaner. I think I was a bit scared because I didn’t think I needed to eat more. I thought that I was eating a lot and I was worried that I had put on weight” (Participant 10—Athlete).

### 3.3. Delivery and Application of Message

The sub-themes and representative quotes associated with the delivery and application of nutrition-related advice are presented in Table 4. The athletes and coaches recognised that enabling and supporting athletes with clear and specific messages to change behaviour is pivotal to increase athlete self-awareness and to make long-term nutrition-related changes. Participant 11 echoed that a specific and prescriptive message on adequate fuelling for health and performance is warranted:

“I’m not saying it has to be completely and utterly prescriptive, but I think sometimes when they’re asked to eat more, they don’t always know what that looks like. A little more explanation into how that might look and an actual visual would help and a little bit more prescription into what it is they need to put in their mouth” (Participant 11—Coach).

Relating the advice on fuelling to athlete performance was also considered a key element in terms of athlete “buy-in”:

“Always trying to bring it back to performance and wellbeing for me was creating myself being (a) more robust athlete. So, if you are more robust and you are healthier, you miss less training. And that comes from eating more. Eating less is just going to have the opposite effect. You are going to be less robust. You are going to be less healthy. And you are more likely of getting sick and injured. Just showing the positive sides of how it will impact their performance is a key thing.” (Participant 15—Athlete).

Athletes described the need for nutritionists to be as specific as possible, and for the athlete themselves to be committed and as organised as possible:

“I think definitely you just have to commit to it because there’s no point to doing it half-assed. Because I think when I started with it, some days I’d be like, “Oh, I’ll have half a shake.” I’ll have a few scoops, but I wouldn’t really be eating more and I didn’t know was they’re actually consuming more calories. So, you just have to commit at the start and know that you’re eating more” (Participant 10—Athlete).

Furthermore, Table 5 outlines athletes’ initial thoughts about consuming more fuel. The thoughts on being advised to consume more calories ranged from being shocked because they thought they ate enough, or worried about weight gain, to acceptance of the advice. Performance nutritionists need to consider these emotions and feelings when advising athletes to fuel adequately. Moreover, athlete participants in this study highlighted that coaches’ reactions to the consumption of more food ranged from positive reinforcement of the nutritional advice:

“There wasn’t any issues. I just let my coach know and it was making me better and training and recovering better and all that. So it was good. He’s glad to have gotten that insight” (Participant 18—Athlete).

To confusion about the additional caloric needs of the athlete:

“The coaches were confused because they were just looking at the numbers of the skinfolds. I think they don’t really understand that the amount of calories that we need to eat during this training. So, I think they just thought that we were all eating a bit too much” (Participant 10—Athlete).

Furthermore, athlete 6 alluded to hesitancy or worry about telling coaches based on coach reaction:

“I’d say they understand that we need to eat 5,000 calories a day, but they might not like the way we do it. But, I’d say the quality of the foods were probably what they’d react to.”

Coaches identified areas where further education is needed: the coach network and parents as well as the athletes themselves. More specifically, preconceived ideas among athletes, the influence of social media, and underestimation of energy and nutrient requirements by athletes require consideration when developing athlete education programmes (Table 6).

## 4. Discussion

The main aim of this study was to explore perceptions towards advice to eat more food to fuel health and performance among athletes and coaches and the challenges that athletes face in implementing this advice. A secondary aim was to explore how nutritionists could deliver advice around fuelling more effectively. The athletes in this study reported improved training consistency, better health outcomes such as fewer injuries and illnesses and improved resilience at training camps due to fuelling adequately. The athletes reporting improved health and performance outcomes received one-to-one nutritional support from a sports nutritionist in Sport Ireland Institute for approximately 6–12 months prior to study commencement. Athletes and coaches reported time management around high training loads and meal preparation as the main challenges faced in fuelling adequately.

Several coaches reported that some of their athletes did not realise that they needed to eat more food to support health and performance. More often the discussion about fuelling for training demands was initiated by the coach or support staff. Athlete reactions to such discussion and advice varied from shock, worry about weight gain, to a moment of realisation whereby the athlete could not believe that they had never thought of eating more themselves to support their training and physiological needs. These findings support the need for athlete, coach, and parent education on the importance of fuelling adequately for health, training, and performance, particularly during the early phases of an athlete’s career to avoid unfavourable health outcomes [3,13]. Some elite athletes and coaches expressed that they were sceptical early in their careers about following advice to consume more calories. However, such advice elicited health and performance benefits that changed their opinions on dietary behaviours such as fuelling adequately.

The distinction between intentional and unintentional under-fuelling is crucial [3,9]. Athletes unintentionally under-fuelling may not be aware of the gap between their energy intake and training. Thus, explaining the health, training, and performance outcomes of LEA is essential for athlete and coach buy-in, especially in terms of relating fuelling to performance, one of the main benefits of eating more identified in this study. The performance nutritionist/dietitian needs to be able to explain the energy requirements for health and training and discuss the physiological signs and symptoms of under-fuelling to ensure that athletes are aware of the negative health and performance outcomes associated with LEA.

Athlete worries and fears surrounding weight and their coaches’ reactions to consuming more fuel were evident in this study: “I think definitely I was just paranoid about gaining weight was my main thing that I struggled with (Athlete—Participant 10); and I’d say they understand that we need to eat 5,000 calories a day, but they might not like the way we do it. But, I’d say the quality of the foods were probably what they’d react to” (Athlete—Participant 6). It is important for sports nutritionists/dietitians to integrate a behavioural change approach whilst working with athletes, and to identify internal and external triggers (e.g., feelings, opinions, or beliefs) that get in the way of planned change [14,15,16]. The way in which the message is delivered is also important for athlete buy-in and to build confidence when making a change [14].

The main challenges to adequate fuelling were time management around training and food preparation in this study. Performance nutritionists should be aware of these challenges, the needs of the sport, and associated training demands. It is evident from our research that performance nutritionists need to provide specific, individualised advice relevant to the individual (for example, school/university timetables, living arrangements, cooking skills/facilities, available budget, likes and dislikes, training times, and phase of training). Visual, prescriptive, individual plans that fully consider key elements/components/factors in the athlete’s life are crucial to helping deliver the “eat more” message, e.g., for low vs high training days.

Furthermore, our findings highlight that additional education is warranted for the medical and support staff, as well as for parents, coaches and athletes themselves: “The perception of what an appropriate and good diet is, is very warped really” (Coach—Participant 1). Although education is important, it is evident from our results that motivating, enabling, and supporting athletes with clear and specific messages to change behaviour is pivotal to increase athlete self-awareness and to make long-term nutrition-related changes [15].

### 4.1. Practical Applications for Performance Nutritionists

The current study findings provide some practical application insights for performance nutritionists. While our findings identified some positive health and performance outcomes following the nutritional advice delivered by a performance nutritionist, it also highlighted a number of improvements necessary to the delivery of the fuelling message by practitioners. Hence, we suggest sports nutrition and dietetic practitioners consider the following practical applications from our findings:

1. Communicate with athletes using visual aids rather than text when demonstrating the quantity of food necessary to fuel health and performance;
2. Interview athletes more frequently (e.g., every two weeks initially) to build rapport and seek feedback to better understand the athletes’ individual needs for adequate fuelling and ensure shared decision-making;
3. Relate the nutritional advice to performance outcomes, e.g., fuelling for training or for better outcomes in training and competition, to highlight the role of nutrition in relation to the short- and long-term performance goals, i.e., allowing athletes to train consistently on a daily basis; and
4. Deliver education evenings or seminars for athletes and coaches to communicate the message in a collaborative or group environment. It may be useful to include other members of the sports science team, i.e., medical and physiotherapy staff, to reinforce the collaborative approach and improve the delivery of the message.

### 4.2. Strengths and Limitations

This qualitative exploration of athletes’ and coaches’ perceptions of fuelling adequately for health and performance has provided novel insights into the benefits and challenges of consuming more food and identified enablers in the delivery of the nutritional advice by dietitians/nutritionists. Another major strength of this study was the inclusion of elite level athletes and coaches. A limitation is that generalisability of our study findings cannot be implied, even within the Irish context. Both athletes and coaches had knowledge of RED-S and access to a sports nutritionist/dietitian in their careers to date. Therefore, the current study findings may not reflect the experiences of other athletes who have experienced or are experiencing daily struggles with consuming enough food for health and performance. Moreover, the use of this small purposive sample also means the benefits and challenges of fuelling adequately reported by our study participants may be less representative of individuals training and competing at an amateur level. Finally, for the purposes of this research study, data on athlete participant energy availability status was not collected. Athlete participants were recruited if they had been advised to consume more calories by their performance nutritionist in Sport Ireland Institute to support their health and performance. Exploration of whether differences of opinion exist based on energy status of the athlete is an area of research that warrants future investigation.

## 5. Conclusions

For the first time, this research provides an overview of the perceptions towards advice to eat more food to fuel health, training and performance among athletes and coaches. The current findings identified fuelling benefits of: (a) more energy; (b) consistency in training; and (c) better recovery, as well as the challenges of: (a) food timing and preparation of food; (b) the volume of food to be eaten; and (c) the financial burden. Enablers in the delivery of the advice to fuel adequately such as: (a) clear communication and collaboration between athlete, coach, and the multidisciplinary team; and (b) prescriptive/individualised nutritional advice that is performance-focused is warranted based on the perspectives of athletes and coaches in this study. Furthermore, these findings suggest that although RED-S education is important, there is a need to motivate, enable, and support athletes to change behaviour. This is pivotal to increase athlete self-awareness and to make long-term nutrition-related change to benefit both health and performance.

## Figures and Tables

**Table 1:** Athlete participant characteristics.

Sports	Elite Level Athletes (*n*)	Age (Years)	Training (Hours)	Competition Level
Athletics	1	30	18	International
Boxing	1	23	20	International
Judo	1	30	25	International
Modern pentathlon	2	26–29	30	International
Rowing	4	22–26	16–40	International

**Table 2:** Coach participant characteristics.

Sports	Coaches (n)	Coaching (Hours)	Coaching Level
Athletics	3	5–8	International
Cricket	1	15	International
Cycling	1	20	International
Rowing	1	30	International
Swimming	3	10–30	International

**Table 3:** Theme: The benefits and challenges of adequate fuelling.

Sub-Theme	Representative Quotes
**Benefits**	
Energy	“Energy, yes. Definitely being able to finish out the session at a high level, rather than nose-diving halfway through the session” (Participant 6—Athlete). “There is a lot more quality coming from each session. I wasn’t going through the motions as much. It was more quality and focused, I guess, during each session. You just have the energy available” (Participant 18—Athlete).
Consistency and better recovery	“So, just kind of overall health being better and just getting more consistency out of training” (Participant 13—Athlete). “Having athletes who are healthy to train everyday consistently, is for me, the biggest beneficial part of this” (Participant 5—Coach).
Overall health and performance	“One of the key things we always try to get across to our athletes, is if you’re not available to train due to illness or sickness then it doesn’t matter if you’re strong as an ox in the gym” (Participant 2—Coach). “I think a big part is that you don’t end up getting little niggles and injuries. Because that kind of comes from when you’re tired or whatever, and then it has a knock-on effect. So, overall health being better and just getting more consistent in training” (Participant 13—Athlete).
**Challenges**	
Timing and preparation of food around training	“Actual time to eat enough can be very difficult, particularly for runners because the mechanical motion of running can make it uncomfortable to eat anywhere close to training. The windows to get food into the athlete are fairly small post training. If an athlete is running twice a day, the window is then reduced to this block just before training. It’s difficult to eat. And then if that’s combined with a work or study schedule, that means there’s not much time between training and in everyday life, that can be really difficult” (Participant 1—Coach). “Huge challenge, trying to just even get the quantity into me and also food preparation was really difficult” (Participant 14—Athlete).
Budget	“Cost for sure, yeah. That is definitely a thing, because if you’re eating well and healthy and you’re trying to get adequate amounts in. There is a cost aspect to that. Good ingredients are going to be obviously more expensive than cheap food, and I think that cost element, depending on where the athlete is at, depending on what level of funds they have available to them” (Participant 9—Coach). “Obviously cost then as well. Your budget goes up as well because you’re buying more and more food. And you want to get good quality food as well. So that’s another factor” (Participant 15—Athlete).
Volume of food	“Just the volume, definitely. It’s a huge volume of food. Timing it. Yeah, the timing and the volume” (Participant 6—Athlete).
Risk of weight gain	“It can be tricky on the weight side. Because when people commit to that new way of eating, sometimes they can put weight on before they lose it, and it gets complex then at that point as to how they need to stick with it longer term. Because it is a change that you see benefit from in the more medium to longer term, it’s not an overnight thing” (Participant 9—Coach).

**Table 4:** Theme: Delivery and application of message.

Sub-Theme	Representative Quotes
Specific and prescriptive	“I think it was really useful to be shown the food, so to see the amount, because I used to make a meal and think that it was fine in calories, but then to actually break it down and it wasn’t. And I think that was really useful to actually visually see what I needed to eat rather than what I thought in my head” (Participant 14—Athlete). “A wishy-washy vague bit of guidance is not good enough, it needs to be really quite prescriptive and forceful, I guess, the advice. Knowing particular personality types of the athletes you’re often dealing with” (Participant 1—Coach). “Stick with it. There will be tough days with eating, especially females I find. I feel like they’ll look at the volume of food or they’ll be like, ‘you will gain weight’. It’s inevitable. But for training, it’s definitely more beneficial in the long run” (Participant 7—Athlete).
Commitment and organisation	“Just be as organised as possible. There is a big gap between what I thought I was getting—I thought I was getting enough and the amount I actually needed to get, like it does, it might seem shocking at first but it really does help” (Participant 18—Athlete).
Communication & Collaboration	“I think it’s making sure that athletes, one, know that there’s a real collective collaborative approach from all the support staff, from the head coach right the way down to the team operations manager” (Participant 2—Coach). “The only advice I sometimes have is to be able to plug into the wider support team around that individual and that athlete. Particularly if you haven’t built a rapport with an athlete” (Participant 8—Coach). “That’s not always communicated fully openly, so it’s to get to know the person and understand their relationship with food, and that might require collaboration with sports psych or someone like that to get a handle on how an individual might respond to information and to advice” (Participant 9—Coach).
Performance-based	“Discussing from a performance benefit point of view is important. I think probably also some athletes need the shock of discussing the long-term implications for their health, if they don’t address it as well. I think there is a sense of urgency of things could go downhill quite quickly” (Participant 1—Coach). “Just always linking it back to the performance, but not just performance in competition, performance in training as well. And just performance in lifestyle as well. The fact that they will have better concentration levels, better focus” (Participant 12—Coach)

**Table 5:** Theme: Initial thoughts on being advised to consume more fuel for health and performance.

Sub-Theme	Representative Quotes
Shock	“Remember being shocked, because I thought I ate enough. I remember being like, ‘Really? But I eat loads.’ So it was definitely a shock” (Participant 14—Athlete). “Shocked. Yeah, little bit. I knew I had to eat more, but I didn’t realise how much more” (Participant 7—Athlete).
Worried about weight gain	“I think I was a bit scared because I didn’t think I needed to eat more. I thought that I was eating a lot and was worried that I’d put on weight” (Participant 10—Athlete). “Yeah, obviously I was kind of like, why? Am I not going to put on more weight, and so on? So, it is obvious there is going to be this kind of a stigma attached to it because I know I’m not a boxer that I have to worry about my weight in that sense. But I still have racing weight” (Participant 15—Athlete).
Accepting/ Penny-drop	“I would say I wasn’t really that shocked, so I kind of knew that I was doing a lot of training, so I had to support it, it was just that maybe I thought that I was getting close enough, but so maybe the gap between what I was having and what I needed to be taking was kind of bigger than I thought, I guess” (Participant 18—Athlete). “I remember just being like, ‘Oh, okay.’ It was just a penny-drop moment. It was things where, for a long time, I have done hypertrophic blocks, I’ve thought that I was eating enough calories. It was just that moment where I was just like, ‘Why have I not thought about that before?’” (Participant 3—Athlete).

**Table 6:** Theme: Gaps in education.

Sub-Theme	Representative Quotes
Perceptions in endurance sport	“There is a perception amongst some particularly older coaches that endurance athletes don’t have a period and that’s normal, it’s part of endurance training. It’s not something that should be a warning sign or flag. It’s accepted as being part of being an endurance athlete” (Participant 1—Coach). “There is a hell of a lot of medics that will still put a young girl on the pill just to bring it back” (Participant 17—Coach).
Underestimation of intake/ ‘clean eating’	“The general society view of what healthy diet is and the notion of a clean diet, etc. I’ve had parents of athletes on national squads where immediately I’ve spent the day with the athlete. I would be concerned that there was an issue and having spoken to the parent, the parent would say, ‘Oh, she eats really well, chicken breasts and a plate full of greens.’ So, the perception of what an appropriate and good diet is, is very warped really. So, there’s multiple challenges and lots of areas of education needed and lots of different groups to be educated” (Participant 1—Coach). “From my experience, we don’t have a problem with the athletes not eating healthy. They eat very healthily. If anything, they eat too healthily” (Participant 16—Coach).
Dangers of social media influences	“I think there is that issue of social media and Instagram and general body image and that is very, very difficult for anybody to get away from” (Participant 2—Coach). “Young athletes are very influenced by what they see, as opposed to what they are told. And unfortunately, it is a hard thing to show. But I think, I mean, I am keeping a little bit of an eye on this movement, because it is a new philosophy and you’re not seeing athletes post pictures of a salad anymore. You see them eating a hamburger, and that is cool. I think that is a very good first step” (Participant 5—Coach). “Like Michael Phelps ate this number of calories a day, this is what he did. He didn’t do it ever. And they don’t actually know what he did. Do you know what I mean? So, that’s a snippet of one day in time over whatever, So I think that’s probably something just to be more mindful of as well. Because I think a lot of the athletes get their information from the social media” (Participant 8—Coach).

## Data Availability

The data presented in this study are available upon reasonable request from the corresponding author, and only after approval by the university ethical committee. The data are not publicly available due to ethical restrictions.
